# Comparing a Tailored Self-Help Mobile App With a Standard Self-Monitoring App for the Treatment of Eating Disorder Symptoms: Randomized Controlled Trial

**DOI:** 10.2196/14972

**Published:** 2019-11-21

**Authors:** Jenna Tregarthen, Jane Paik Kim, Shiri Sadeh-Sharvit, Eric Neri, Hannah Welch, James Lock

**Affiliations:** 1 Recovery Record San Francisco, CA United States; 2 Stanford University School of Medicine Stanford, CA United States

**Keywords:** mobile health, smartphone, mobile apps, eating disorders, cognitive behavioral therapy, mental health, intervention study

## Abstract

**Background:**

Eating disorders severely impact psychological, physical, and social functioning, and yet, the majority of individuals with eating disorders do not receive treatment. Mobile health apps have the potential to decrease access barriers to care and reach individuals who have been underserved by traditional treatment modalities.

**Objective:**

The objective of this study was to evaluate the effectiveness of a tailored, fully automated self-help version of *Recovery Record*, an app developed for eating disorders management. We examined differences in eating disorder symptom change in app users that were randomized to receive either a standard, cognitive behavioral therapy–based version of the app or a tailored version that included algorithmically determined clinical content aligned with baseline and evolving user eating disorder symptom profiles.

**Methods:**

Participants were people with eating disorder symptoms who did not have access to traditional treatment options and were recruited via the open-access *Recovery Record* app to participate in this randomized controlled trial. We examined both continuous and categorical clinical improvement outcomes (measured with the self-report Eating Disorder Examination Questionnaire [EDE-Q]) in both intervention groups.

**Results:**

Between December 2016 and August 2018, 3294 *Recovery Record* app users were recruited into the study, out of which 959 were considered engaged, completed follow-up assessments, and were included in the analyses. Both study groups achieved significant overall outcome improvement, with 61.6% (180/292) of the tailored group and 55.4% (158/285) of the standard group achieving a clinically meaningful change in the EDE-Q, on average. There were no statistically significant differences between randomized groups for continuous outcomes, but a pattern of improvement being greater in the tailored group was evident. The rate of remission on the EDE-Q at 8 weeks was significantly greater in the group receiving the tailored version (*d*=0.22; *P*≤.001).

**Conclusions:**

This is the first report to compare the relative efficacy of two versions of a mobile app for eating disorders. The data suggest that underserved individuals with eating disorder symptoms may benefit clinically from a self-help app and that personalizing app content to specific clinical presentations may be more effective in promoting symptomatic remission on the EDE-Q than content that offers a generic approach.

**Trial Registration:**

ClinicalTrials.gov NCT02503098; https://clinicaltrials.gov/ct2/show/NCT02503098.

## Introduction

### Background

The need for scalable delivery of eating disorder (ED) care services that are clinically effective and broadly accessible is now a major public health priority. EDs are common mental disorders that are both psychologically debilitating and physically threatening. Approximately 13% of young women and 1.93% to 6% of adults will meet the criteria for an ED in their lifetime, and 3% to 3.5% of men also struggle with an ED [1-4]. Despite the severity and burden of EDs, they often remain undetected, and the majority of individuals with EDs do not seek or receive mental health care [5,6]. Recent systematic reviews found that as few as 23% of people with a diagnosable ED seek conventional treatment [5], and about only 1 in 10 individuals with this illness receive treatment [7].

There are significant barriers to access to ED treatments, including high cost of care, inadequate insurance coverage [8,9], paucity of trained clinicians [10,11], and experiences of shame or fear of stigmatization [12]. One study found that it took individuals, on average, 3.6 years to acknowledge that they were suffering from an ED and a further 4.2 to 6.3 years to seek treatment [13]. Unfortunately, these delays are costly, as over time, EDs become more severe and less responsive to treatment [12,14]. There is evidence that the duration of ED is adversely associated with the treatment outcome [15,16]. However, even if all people with EDs were to seek conventional treatment, the current models of treatment delivery would be insufficient to meet the enormous need. A major shift in intervention practice is warranted with a focus on reaching more individuals in a more cost-effective manner, while at the same time achieving clinically meaningful improvement. Mobile health (mHealth) apps will almost certainly play a role because of their reach and breadth of functionality.

At least 271 million people in the United States or 94% of the population own a mobile phone, and smartphone use has reached 77% population penetration, with uptake spanning all socioeconomic groups [17]. mHealth apps have the potential to decrease the aforementioned treatment access gap for EDs and reach individuals who have traditionally been underserved by existing treatment modalities. By offering anonymous, accessible, affordable, and engaging interventions, barriers to receiving care can be reduced. The convenience of an intervention that can be accessed in moments of need at any location may enhance acceptability, and the scalable nature of technology holds promise for delivering support in a cost-effective manner [18].

### Objectives

One example of an mHealth app for EDs is Recovery Record (RR). RR has established population-level reach and user acceptability [19]. Although RR was initially developed as an adjunctive tool to support clinical treatment, a large portion of app users access the tool without the accompanying forms of traditional face-to-face treatment. A 2014 case report surveyed over 100,000 RR app users and found that 46% were not receiving clinical treatment, and 33% of users reported that they had not told anyone about their ED [19]. The study further found that 80% of users had experienced symptoms for 5 to 10 years, and 58.3% had Eating Disorder Examination Questionnaire (EDE-Q) global scores of 2 or more SDs above community norms [20]. Hence, the RR app was found to be successful at reaching and engaging many people with severe and enduring ED symptoms who were otherwise not receiving care.

Incorporated into RR app’s core functionality are cognitive behavioral therapy (CBT)–based eating and symptom monitoring, CBT-style coping skills, goal setting, and motivational messaging. Self-help CBT can be an effective intervention for some EDs, and preliminary data suggest that RR might be effective as a stand-alone self-help intervention. Data from 1178 RR app users who were not receiving clinical treatment revealed that after using RR for 1 month, 28% of participants no longer scored in the clinical range on the EDE-Q and 39% were clinically improved [21]. These response rates approximate those observed in studies of therapist-assisted internet-based treatments for EDs [22,23]. Another study found that RR users naturally clustered into 5 clinical groups that could be mapped onto the existing Diagnostic and Statistical Manual of Mental Disorders ED categories [24]. Of further interest, a signal detection analysis revealed that RR intervention response was not homogenous across the sample and that outcome varied by clinical presentation. For example, those with binge eating and purging symptoms were found to be more likely to respond to the RR app than those with mostly restrictive behaviors [21].

Overall, these data indicate that there are distinct RR user groups who already utilize the app and may derive greater clinical benefit from a personalized intervention that targets their specific clinical needs [25]. As a next step, a new tailored version of the RR app was developed, including an 8-week program of personalized content specifically addressing baseline and evolving clinical characteristics. A pilot study demonstrated the feasibility of deploying the tailored version of the app to a sample of 189 app users and validated acceptability of the new intervention developed by the study team [26].

The purpose of this study was to examine whether a personalized app for EDs would be superior to the universal app in reducing negative outcomes when used in self-help capacity. Specifically, we were interested in studying the differences in symptom change in users of RR that were randomized to either the standard RR app (RR-S) or the tailored version of RR (RR-T), which included algorithmically determined content aligned with user baseline ED symptom profiles. Our primary hypothesis was that those who received RR-T would demonstrate greater clinical improvements compared with those who received RR-S.

## Methods

### Participants

RR app is free and publicly available via the Google Play (Android) and iTunes (iPhone) app stores. Potential participants were recruited from within the app registration system. All users were asked to provide consent. Users were eligible for inclusion if they (1) had downloaded the app on their iPhone, (2) were located in the United States, and (3) recorded at least three self-monitoring entries before being contacted about the study. The focus of this study was on underserved populations who might not have access to best practice treatment options. As such, individuals were considered ineligible to join the study if they were using RR linked with a treatment provider or indicated that they were receiving treatment at least weekly from a specialist ED provider. The study received Institutional Review Board approval, and participants did not receive any payment for completing assessments.

### Study Design

#### Randomization

Participants randomized to RR-T were probabilistically assigned to 1 of the 5 clusters based on their baseline demographic characteristics and EDE-Q scores. Each participant was randomly assigned to a cluster with a probability inversely proportional to his or her distance to each cluster mean. This distance was defined as the Euclidean distance between a participant’s coordinates (ie, all baseline measures) and the cluster mean. This method meant that participants were more likely to be assigned to the symptom cluster they were most similar to.

#### Tailored Intervention

**Figure 1:**
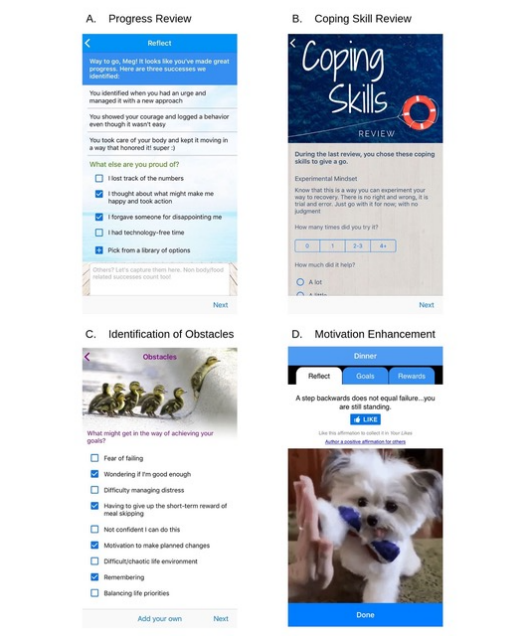
Select recovery record adaptive application features.

Details on the app and the development of the tailored intervention have been described in earlier reports [24,26]. Informed by baseline cluster assignment and existing knowledge about CBT-based strategies for addressing ED symptoms and cognitive distortions, novel and tailored content was developed for each baseline symptom cluster group. Descriptions and examples of each key feature are provided in Table 1. The tailored intervention took the form of an 8-week program that delivered tailored content to complement the standard app. Specifically, the tailored app is configured with cognitive behavioral self-monitoring questions that are differentiated according to user baseline symptom cluster assignment. Users in the tailored group were also invited to complete a progress review on a weekly basis. Components of the progress review included the following: a summary of recovery-oriented milestones achieved (see Figure 1); a self-guided review of goal progress and perceived helpfulness of coping skills (see Figure 1); the selection of new goals and coping strategies from a curated, tailored list for the week ahead; and, finally, the identification of possible obstacles to achieving chosen goals (see Figure 1).

The weekly goal selection was designed to encourage task practice of specific activities each day and then to facilitate rating of activities on the degree of mastery and/or pleasure in the weekly progress review. Goal options follow the CBT for EDs framework and aim to disrupt mechanisms that may be maintaining symptoms reflected in baseline profiles and ongoing meal logs. Skill-based components of the tailored version of the app provide the opportunity to learn and implement strategies for managing distorted cognitions that fuel both the emotional and behavioral responses to engage in unhealthy eating or weight loss practices. Given the limited presentation capacity of a smartphone, content is skill- and goal-based rather than psychoeducational and aims to maximize user engagement and generalizability rather than present large amounts of data.

**Table 1:** Key Recovery Record tailored app features.

Feature	Description	Example
Customized self-monitoring questions	Self-monitoring questions are customized based on baseline symptoms. Participants can also optionally enable additional questions if relevant to their needs.	If a participant endorsed binge eating in their baseline questionnaire, then the questions “Did you binge eat?” and “Do you have an urge to binge eat?” are included in meal logs.
Weekly milestones	Each week the app displays 4 to 7 user achievements based on participants’ daily self-monitoring entries. Participants can also optionally enter additional achievements not captured by the app.	If a participant indicated in a meal log that they were experiencing an urge and, in the same entry, endorsed the use of a coping strategy, the following weekly milestone would be displayed: “You discovered <number> new coping strategies for responding to a difficult feeling or urge.”
Goal progress review	On a weekly basis, the app displays the SMART^a^-style goals that the participant had selected in the prior week and prompts them to evaluate goal progress.	If a user had previously selected a goal to preplan their meals, they would be asked how they are progressing toward the goal, with the following response options: “I haven’t thought about it yet,” “I have thought about it,” “I have a plan and will put it into action today,” “I did this several days this week,” and “I did this every day.”
Coping skill review	Following the goal progress review, the app displays coping skills selected in the prior week and prompts the participants to evaluate their utility and helpfulness.	If a user had selected “Mindful Eating” in the prior week, they would be asked how many times they tried the technique, with 0, 1, 2 to 3, and ≥4 response options, and to evaluate how much the skill helped on a Likert scale.
Weekly goal selection	An 8-week program of SMART-style goals was developed for each baseline symptom cluster group. Each week, 4 to 6 goals are presented to the participants who are invited to select at least two goals to work on each day of the upcoming week. Users are prompted on a daily basis during the week, at a time they select, to review their progress.	If a user has baseline dietary restriction symptoms, they may be presented with the optional goal to keep track of their triggers: “I will notice and record dietary restriction triggers in Recovery Record. To identify triggers, I will ask, ‘what set me off?’ Triggers amplify eating disordered thinking and make me more vulnerable to relapse. Examples: Feeling unwell, drinking alcohol, certain emotions, body comments, negative self-talk, weight gain, confrontation, financial stress, lack of sleep.”
Weekly coping skill selection	An 8-week program of coping skills was developed for each baseline symptom cluster group to complement the program of goals. Each week, 4 to 6 coping skills are presented to the participants who are invited to select at least two skills to try out in the upcoming week. Users are prompted to utilize their selected skills in real time when they self-monitor relevant symptoms.	If a participant has baseline binge eating symptoms and intrusive thoughts, they may be presented with the “Questioning the Evidence” skill to: “Catch the actual thoughts you are thinking when you’re in a situation that upsets you. Examine them to see if they’re valid. Ask: Where’s the evidence for this? What do you get if you ‘buy’ into that thought? Where does it leave you and does it bring you closer to your best self? Consider these example thoughts: ‘If I keep X food in the house, I can prove I am strong enough to recover,’ ‘My eating problem has already ruined X,’ ‘What do I have to gain from recovering now?’.”
Obstacle identification	A list of potential barriers or obstacles that participants may experience when trying to achieve their goals is presented. Participants select obstacles that are relevant to them and identify actions they can take to overcome them.	If a participant selected a goal of eating something at every meal and snack, a suggested barrier to action might be “Having to give up the short-term reward of meal skipping.”

^a^SMART: 8-week program of coping skills for each symptom cluster group.

#### Standard App Intervention

Users randomly assigned to the standard app were also prompted to complete meal and symptom self-monitoring in an evidence-based CBT format that has been described previously [19]; however, they did not have access to the weekly progress review, including tailored milestone feedback, coping skill and goal content, or obstacle identification. Both versions of the app also included psychoeducation regarding skills to increase distress tolerance and overcome urges to engage in disordered behaviors and included textual and image affirmation content targeting motivational enhancement (see Figure 1).

#### Clinical Outcomes

The EDE-Q is a self-report measure of ED psychopathology and behaviors that has been shown to have good reliability [20,27]. We examined both continuous and categorical outcomes related to clinical improvement in ED psychopathology in the randomized groups at baseline, 4 weeks, and 8 weeks. At the relevant time intervals, participants were prompted with a banner on the home screen within the app to complete the in-app EDE-Q assessment. An automated email was also delivered to participants to notify them when an assessment was available within the app.

#### Primary Outcome

The primary dichotomous outcome of a response, that is, clinically meaningful change, was defined as an improvement (ie, decrease) in the EDE-Q global score by a 0.5 SD. A secondary outcome of remission on the EDE-Q was defined as being within the range of 1 SD around the mean, based on the global EDE-Q (community norm of 1.55) [20].

#### Secondary Analysis

Frequencies of objective binges, vomiting, and excessive exercise over the previous 28 days were derived from EDE-Q questions 14, 16, and 18, respectively. The categorical outcomes for abstinence were defined as whether the participant endorsed 0 instances of binge eating (or purging or excessive exercise) at follow-up. We also examined continuous outcomes defined as the differences in the EDE-Q item 14 between baseline, week 4, and week 8. We repeated this outcome analysis using items 16 and 18 on the EDE-Q.

### Statistical Analysis

#### Primary Analysis

To address the primary hypothesis that RR-T improves EDE-Q total score, a complete case analysis was used. All participants randomized to the 2 treatment conditions and who had outcome data (week 4 or 8) were included in the analysis. To determine whether a clinical improvement in the RR-T arm occurred at 4 and 8 weeks, two-sample *z* tests for proportions were used. Effect sizes (ie, success rate differences) were reported. All tests were 2-sided and performed at the 0.05 level of significance.

We note that complete case analysis will only be unbiased under the missing completely at random (MCAR) assumption, that is, it is valid only when the missingness probability does not depend on the outcome [28].

Covariate adjustment was performed to address a secondary hypothesis of whether there was conditional independence between the treatment assignment and clinical improvement, given other variables, that is, we tested a secondary hypothesis of whether there was a treatment effect *within* strata defined by the variables mentioned above. This covariate adjustment analysis addresses a different null hypothesis than the primary hypothesis of testing the unconditional treatment effect. Covariate adjustment was performed using generalized linear mixed models and linear mixed models as appropriate, with the treatment assignment indicator, treatment by time interaction, and other variables including baseline severity and duration of app usage. Gender and treatment frequency were not used because of sparsity in groups.

#### Secondary Analysis

A sensitivity analysis was conducted using clinical end points defined by a change in EDE-Q global score by 0.75 SD and by 0.25 SD. We conducted an analysis using the outcome of remission as defined above. Outcomes of remission were binary and remission rates, that is, proportions were computed for each arm at each time point. Differences between the remissions rates observed in RR-T and RR-S arms at weeks 4 and 8 were evaluated by *z* tests for proportions, with a significance level of 0.05. We also constructed graphical summaries of the proportion of remitters over time per arm.

A per-protocol analysis was performed, excluding subjects who failed to submit logs over a duration of less than 35 days (out of 69 possible days). The threshold for the inactive period, that is, 35 days, was determined via exploratory data analysis including histograms. To determine whether a clinical improvement in the RR-T arm occurred at 4 and 8 weeks as the clinical end points, *z* tests for proportions were used. All tests were 2-sided and performed at the 0.05 level of significance.

Subgroup analyses were performed for ED behaviors such as objective binge eating, vomiting, and excessive exercise as indicated by items 14, 16, and 18 on the EDE-Q, respectively. We performed a subgroup analysis among participants who endorsed nonzero instances of binge eating, purging, and excessive exercise, as indicated by items 14, 16, and 18, respectively, on the EDE-Q at baseline. Participants who did not endorse such behaviors at baseline were excluded from this analysis. To compare proportions of abstainers across randomized groups, an intention-to-treat (ITT) analysis was used. To determine whether group differences in eating behaviors (with respect to binge eating, purging, and over exercise) occurred at 4 and 8 weeks, *z* tests for proportions were used. Proportions of individuals who experienced a worsening of the raw global EDE-Q score were assessed at weeks 4 and 8. It should be noted that in the absence of a known cut point for clinically meaningful negative change in the EDE-Q global score, any negative directional change in this score was included in this portion of the analysis.

## Results

### Sample Characteristics

**Figure 2:**
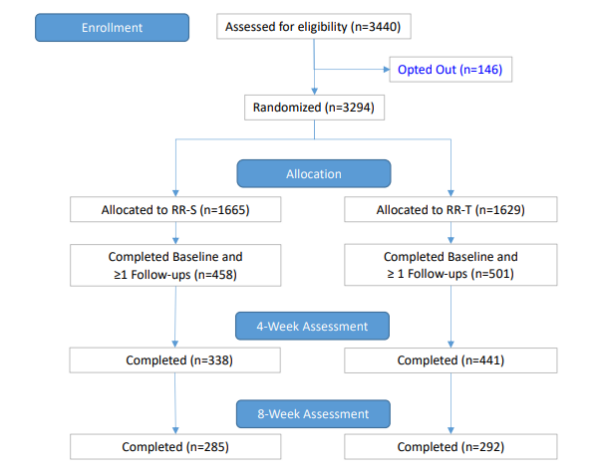
Consolidated Standards of Reporting Trials diagram. There were 15 excluded (13 RR-S and 2 RR-T) at 4 weeks because their EDE-Q was completed outside of a 7-day window from the expected completion at day 30. There were 39 excluded (6 RR-S and 33 RR-T) at 8 weeks because their EDE-Q was completed outside of a 7-day window from the expected completion at day 60. EDE-Q: Eating Disorder Examination Questionnaire; RR-S: standard Recovery Record app; and RR-T: tailored version of Recovery Record app.

A total of 3440 RR users met eligibility criteria between the months of December 2016 and August 2018 and were invited to complete an in-app EDE-Q self-assessment as per current procedure (see Figure 2 for a Consolidated Standards of Reporting Trials diagram). Of these, 146 declined to participate in the study, leaving 3294 who were randomized: 1665 participants were randomized to the standard, fully automated self-help intervention (RR-S) and 1629 participants were randomized to the personalized, tailored self-help intervention (RR-T). Chance imbalances in the randomized group numbers are attributable to our use of a simple randomization procedure. A total of 123 participants reported 1 of the following exclusion criteria after the start of the trial: dizziness, hospitalization, fainting, or suicidal ideation—requiring them to be withdrawn from the study. There were 15 participants (13 RR-S and 2 RR-T) who were excluded at 4 weeks because their EDE-Q was completed outside of a 7-day window from the expected completion at day 30. There were 39 participants (6 RR-S and 33 RR-T) who were excluded at 8 weeks because their EDE-Q was completed outside of a 7-day window from the expected completion at day 60.

Table 2 presents a summary of demographic and usage characteristics of the sample at week 4. All demographic characteristics were balanced between groups. Moreover, 93% (426/458) participants in the standard group were female and 95% (455/501) participants in the tailored group were female, with 4.6% (21/458) [2.6% (13/501) tailored] reporting male gender and 2.4% (11/458) [2.2% (11/501) tailored] reporting *other*. The mean age of the participants was 34 (SD 12.3) years in the standard group and 34.9 (SD 12.5) years in the tailored group. Quartiles of the global EDE-Q score were all severe (Quartile 1: [0.35, 3.12]; Quartile 2: [3.12,3.84]; Quartile 3: [3.84,4.58]; Quartile 4: [4.58,5.95]).

**Table 2:** Demographic characteristics of participants.

Demographical descriptors		Standard Recovery Record app (RR-S; n=458)	Tailored version of Recovery Record app (RR-T; n=501)
Age (years), mean (SD)		34.0 (12.3)	34.9 (12.5)
**Gender, n (%)**			
	Female	426 (93.0)	477 (95.2)
	Male	21 (4.6)	13 (2.6)
	Other	11 (2.4)	11 (2.2)
**Race or ethnicity, n (%)**			
	White	385 (84.1)	407 (81.2)
	Hispanic or Latino	14 (3.1)	22 (4.4)
	Asian	13 (2.8)	20 (4.0)
	Black or African American	13 (2.8)	13 (2.6)
	American Indian or Alaska Native	1 (0.2)	1 (0.2)
	Multiple race or ethnicity	29 (6.3)	22 (4.4)
	Unknown	3 (0.7)	16 (3.2)
**Eating** **problem—how long? (years), n (%)**			
	0	19 (4.1)	20 (4.0)
	1-5	130 (28.4)	113 (22.6)
	6-10	80 (17.5)	102 (20.4)
	11-15	57 (12.4)	58 (11.6)
	15-25	70 (15.3)	90 (18.0)
	≥25	102 (22.3)	118 (23.6)
Body mass index^a^, mean (SD)		29.0 (8.9)^b^	28.7 (8.6)^c^
**Treatment history, n (%)**			
	I have never received treatment for an eating disorder	239 (52.2)	249 (49.7)
	I have received treatment for an eating disorder in the past	145 (31.7)	169 (33.7)
	I am currently receiving treatment for an eating disorder	74 (16.2)	83 (16.6)
**Treatment frequency (for those currently receiving treatment for an eating disorder), n (%); (N=74 RR-S, N=83 RR-T)**			
	2-3 times per month	46 (62.2)	57 (68.7)
	Monthly or less	19 (25.7)	19 (22.9)
	Occasionally or as needed	9 (12.2)	7 (8.4)
**Treatment types (participants could choose more than one; N=74), n (%); (N=74 RR-S, N=83 RR-T)**			
	Licensed mental health professional	64 (86.5)	66 (79.5)
	Dietitian or nutritionist	36 (48.6)	46 (55.4)
	Life coach or mentor	2 (2.7)	5 (6.0)
	Support group or advocacy organization	9 (12.2)	13 (15.7)
	Other	5 (6.8)	5 (6.0)

^a^Excluded 2 standard and 3 tailored Recovery Record app participants with body mass index >65.

^b^n=427.

^c^n=469.

### Analyses

#### Unadjusted Analysis

**Figure 3:**
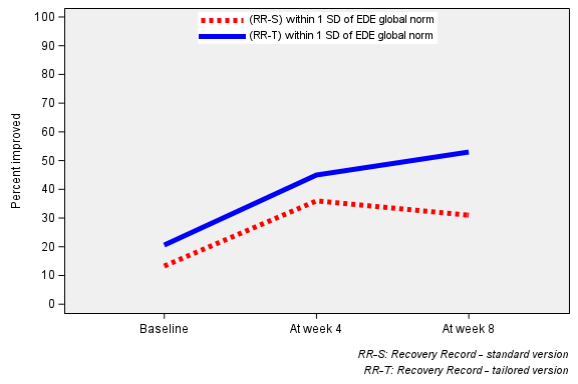
Proportions of responders at weeks 4 and 8. EDE: Eating Disorder Examination; RR-S: standard Recovery Record app; RR-T: tailored version of Recovery Record app.

The responder proportions in the tailored and standard groups were moderately large. At week 4, approximately half (51.5%; 227/441) of the tailored group achieved a clinically meaningful change in EDE-Q, compared with 46.2% (156/338) of the standard group. At week 8, the proportion of treatment responders was slightly greater, with 61.6% (180/292) of the tailored group achieving a clinically meaningful change, compared with 55.4% (158/285) of the standard (see Figure 3). Responder proportions were not statistically different across treatment and control groups at week 4 or 8 (*P*=.16 or *P*=.15; effect sizes=0.05 and 0.06, respectively). Both groups experienced slight improvements in the global EDE-Q score from baseline to week 4 (−0.8 and −0.7 for treatment and control groups, respectively) and from baseline to week 8 (−0.99 and −1.0 for treatment and control groups, respectively).

#### Sensitivity Analysis

We repeated the unadjusted analysis replacing the outcome of clinically meaningful change based on a 0.25 SD change and 0.75 SD change, respectively. Figure 3 presents the sensitivity analysis. There were no statistically significant differences between randomized groups found in this analysis.

#### Covariate Adjustment

The covariate-adjusted treatment effect is consistent with the ITT analysis (conditional odds ratio [OR] 1.2; *P*=.59). Results from generalized linear mixed-effects model estimates showed that subjects with a higher baseline severity (>3 global EDE-Q) were more likely to achieve a clinically meaningful change (conditional OR 3.5; *P*<.001). Although treatment and comparison groups did not differ over time, the effect of time was significant (conditional OR 1.12; *P*=.01): users were 12% more likely to achieve improvement for each additional week of being in the study, holding group assignment constant.

#### Remission Analysis

**Figure 4:**
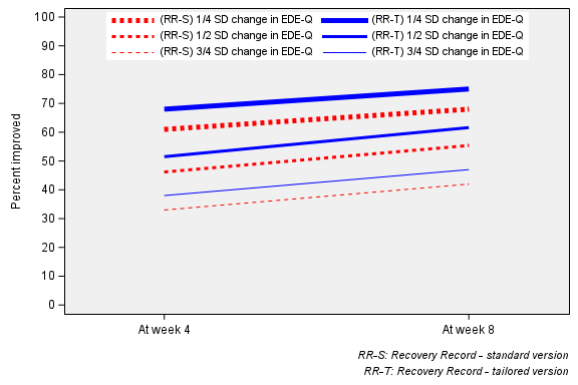
Proportions of individuals whose EDE-Q scores were within community norms at weeks 4 and 8. EDE-Q: Eating Disorder Examination Questionnaire; RR-S: standard Recovery Record app; RR-T: tailored version of Recovery Record app.

Figure 4 presents the remission analysis. At week 4, the proportions of users reporting symptoms within community norms in both groups increased; however, the difference between groups also widened: 44.8% (198/441) of participants receiving the tailored app were remitters, and 35.5% (120/338) of participants receiving the standard app were remitters (*P* value for *z* test of proportions=.008; effect size=0.09). At week 8, the proportion of participants receiving the tailored app meeting the community norms criteria increased to 53.3% (137/257), whereas that of participants receiving the standard app slightly decreased to 31.1% (70/225; *P* value for *z* test of proportions ≤.001; effect size=0.22).

#### Per-Protocol Analysis

Among the tailored group, 57% (166/290) achieved a clinically meaningful change in EDE-Q at week 4, compared with 48% (47/98) in the standard group (*P*=.16; effect size=0.09). At week 8, the proportion of responders was slightly greater, with 63% (138/219) of the tailored group achieving a clinically meaningful change, compared with 53% (62/118) of the standard (*P*=.08; effect size=0.10).

#### Subgroup Analyses

Table 3 presents the proportions of abstainers. At baseline, the number of participants who endorsed any binge episodes did not vary significantly by group: 1390 participants in the tailored versus 1407 in the standard arm endorsed some binge eating (tailored=409 and standard=422 endorsed purging; tailored=705 and standard=753 endorsed excessive exercise). At week 4, the proportion of abstainers for binge eating was 14% (51/359) and 13% (38/287) of the tailored and standard groups, respectively. For purging, abstainers comprised 28% (27/96) and 35% (28/81) of the tailored and standard groups, respectively. For excessive exercise, higher proportions were observed— 40.6% (73/180) and 29.5% (44/149) in the tailored and standard groups. At week 8, the proportion of abstainers slightly increased with respect to binging [20% (49/241) vs 18% (40/227)] and purging [42% (21/52) vs 40% (26/64)], but the proportion of abstainers for excessive exercise decreased [45% (47/104) vs 40% (47/116)]. There were no significant differences between groups on any of these variables.

**Table 3:** Eating behaviors of subgroups of participants who endorsed eating behaviors at baseline.

Eating behaviors			Standard Recovery Record app		Tailored version of Recovery Record app		*P* value
			Total number of participants	Values^a^	Total number of participants	Values	
**Week 4**							
	**Objective binge^b^**						
		Abstinent^c^, n (%)	359	51 (14.2)	287	38 (13)	.81
		Change in score^d^, mean (SD)	359	−4.3 (9.4)	287	−4.2 (10.8)	.87
	**Purge^e^**						
		Abstinent, n (%)	96	27 (28.1)	81	28 (34.6)	.41
		Change in score, mean (SD)	96	−2.0 (10.3)	81	−2.8 (6.4)	.55
	**Objective binge and purge**						
		Abstinent, n (%)	373	47 (13)	295	37 (13)	.99
		Change in score, mean (SD)	373	−4.4 (12.1)	295	−4.8 (11.4)	.67
	**Excessive exercise^f^**						
		Abstinent, n (%)	180	73 (40.6)	149	44 (29.5)	.05
		Change in score, mean (SD)	180	−5.1 (2.9)	149	−3.4 (2.4)	.12
**Week 8**							
	**Objective binge**						
		Abstinent, n (%)	241	49 (20.3)	227	40 (17.6)	.37
		Change in score, mean (SD)	241	−5.5 (11.0)	227	−7.0 (9.8)	.11
	**Purge**						
		Abstinent, n (%)	52	21 (42.0)	64	26 (40.6)	.99
		Change in score, mean (SD)	52	−4.6 (7.9)	64	−3.7 (8.1)	.54
	**Objective binge and purge**						
		Abstinent, n (%)	249	48 (19.3)	238	42 (17.6)	.71
		Change in score, mean (SD)	249	−6.0 (11.7)	238	−7.6 (11.5)	.14
	**Excessive exercise**						
		Abstinent, n (%)	104	47 (45.2)	116	47 (40.5)	.57
		Change in score, mean (SD)	104	−5.0 (7.9)	116	−4.4 (7.7)	.57

^a^Values: Values refer to “n (%)”; or “mean (SD)” as appropriate.

^b^Objective Binge: participant report of eating what other people would regard as an unusually large amount of food and experiencing a sense of loss of control while eating.

^c^Abstinent: participants who abstained from behavior.

^d^Change in Score: the difference in the binge (or purge) items from the EDE-Q questionnaire.

^e^Purge: participant report of making oneself sick (vomit) as a means of controlling shape and weight.

^f^Excessive exercise: participant report of exercising in a “driven” or “compulsive” way as a means of controlling weight, shape or amount of fat or to burn off calories

### Worsening of Pathology in Terms of Raw Eating Disorder Examination Questionnaire Global Score

At week 4, we observed that 16% (59/374) of the tailored group experienced a directional worsening of raw EDE-Q global score, compared with 23% (67/296) of the standard group (*P*=.03, before multiple comparison correction). After correcting for multiple comparisons, the difference at week 4 was not significant. At week 8, 15% (39/250) of the tailored group experienced a directional worsening of raw EDE-Q global score, compared with 19% (47/238) of the standard group (*P*=.28). In the absence of a known cut point for clinically meaningful negative change in the EDE-Q global score, any negative directional change in this score was included in this portion of the analysis.

## Discussion

Individuals with EDs are in urgent need of more affordable, accessible, empirically supported, and engaging interventions. This study is important because it is the first randomized controlled trial to evaluate the efficacy of a personalized app for the self-management of EDs. The study makes an important contribution to the field in its focus on an under-researched and underserved population—people with ED symptoms who may not otherwise have access to traditional treatment options.

### Principal Findings

Although there were no statistical differences (including in the sensitivity analyses) between randomized groups for continuous outcomes, the pattern of improvement was greater in the personalized, tailored version of the app. However, participants in both the tailored and standard app groups achieved a high overall level of response, with more than 50% of participants in each group achieving clinically meaningful change on the EDE-Q at week 8. These response rates indicate that both versions of the app may be beneficial. It should be noted that as yet, there is no standard definition of clinically meaningful change in EDE-Q global scores [29]. As such, a moderate effect size was utilized in this primary analysis.

When examining remission status on the EDE-Q as a categorical outcome, we detected a statistically significant difference between the groups associated with a small effect size. In this study, remission was defined as a score within 1 SD of the community norm, which suggests that symptoms are no longer in the clinical range. These results are encouraging as many app users do not have access to therapists or other treatments, and the tailored version moves more of them out of the clinical range than the standard app.

Contrary to previous research findings [21], we did not find substantial evidence that individuals with mostly restrictive behaviors are less likely to respond to the RR app. Given the transdiagnostic approach to EDs, adults with restrictive symptoms may benefit from a CBT-focused app [30]. This is an important contribution to the literature, given that there are very few studies of self-help for anorexia nervosa. Clinical improvement instead appeared to be related to symptom severity. Participants with higher baseline severity were more likely to achieve clinically meaningful change. It is noteworthy, however, that according to baseline EDE-Q scores, the sample as whole was extremely ill. Therefore, although clinical change was greatest in the most severe group, it might be less dramatic in the groups, overall [30]. It is also possible that there are attributes of participants with high symptom severity not captured in this study that moderated outcome. We also examined changes in objective binge eating, purging, and exercise in the 2 randomized groups (see Table 3). There were improvements in these behaviors across the sample, with no differences between the 2 groups. Finally, we examined whether some participants worsened using the app. We found that approximately 15% to 20% of the participants experienced a directional worsening of their EDE-Q global score during the study app, with no differences between the groups. In the absence of validated negative change cut point for the EDE-Q scores, it is difficult to determine what portion of these individuals experienced clinically meaningful deterioration.

### Strengths and Limitations

Several novel aspects of the study should be emphasized: design of the intervention components; use of a tailored randomization scheme for the *tailored arm*, that is, probabilistically assigning people to clusters; naturalistic recruitment within the app’s existing user pool; and all screening, recruitment, randomization, and assessments being completed within the app. Nevertheless, there are significant limitations of the study. As the intervention is disseminated through an app, our study inherits a host of challenges that come with the large-scale usage of mobile devices in intervention research. Among the challenges addressed during the study were the implementation of the intervention and recruitment of nonpatient participants, strategies to assess compliance and engagement, and problems related to study retention in the absence of the accountability that in-person recruitment affords.

Although we attempted to obtain complete records to the extent possible through the delivery of reminder emails and a lottery for a gift card, it should be noted that of those who were initially randomized, 23.6% (779/3294) provided outcome data at week 4 and even fewer at week 8 (577/3294, 17.5%). Given the high proportion of missing data, an imputation approach would have forced the reliance on an imputation model for 67% of the data and thus presented an infeasible option. To handle the missing data issue, we used a complete case analysis (see the study by Little and Rubin [28] for more details on this approach). A limitation of the complete case analysis is that the unbiasedness of a complete case analysis is predicated on the validity of the MCAR assumption. Although not without limitations, we deemed that it was the most reasonable analytic strategy, given the percentage of data observed. This limitation should be noted in our instance, and the inferences are based on a subset of participants who adhered to the assessment completion. This result, in fact, provided a point to consider for future work, in that a brief period of app usage assessment before randomization should be incorporated in other or future randomized studies to address this data problem.

With regard to study retention, there is a known high variability of dropout rates in studies using self-help treatments for EDs, ranging from 0% to 62% [31]. High dropout rates are common in patients with EDs, even in face-to-face therapy [32]. Within-app recruitment may have additionally contributed to attrition and/or lack of adherence during the study, although attrition rates did not differ based on treatment allocation. These challenges may also have been related to the population of interest, that is, individuals lacking in adjunctive support structures outside of the app. Therefore, the attrition rate observed in this study should not be surprising, considering the in-app recruitment and realities of smartphone app compliance [33]. In fact, the observed rate could offer a perspective to the literature as it provides evidence for appropriate and realistic considerations for power that should be taken into account at the early stages of study design. We should note that we accounted for the probability of a high attrition rate when designing the study, such that our resultant power calculations were based on the number needed in each group to detect a difference of at least 80% power.

Another important limitation is that follow-up data on maintenance of treatment effects is limited because of the short 8-week follow-up period. The effect of time was significant in the study, with users 12% more likely to achieve improvement for each additional week of being in the study. This raises the question of optimal intervention duration. Future studies should aim to assess duration of treatment effects and whether this relates to user characteristics such as symptoms, severity, demographics, motivation or compliance, and/or app content during the intervention period. Diversity across ethnic groups represented in the sample was a limitation of the study. An additional avenue for future research may be to explore the relative effect of ethnicity on outcome.

### Conclusions

The results of this study suggest that a significant proportion of ED app users benefit from using a self-help version of the RR app; however, overall clinical improvements may be greater and symptomatic remission may be significantly greater, with a more specific matching of content to specific clinical groupings as in the tailored version of the app used in this study. More research should be conducted on how app-based self-help can be integrated into a stepped care model of ED interventions, thereby closing the treatment gap. The results suggest that apps that use tailored contents are feasible to use; likely effective for many in improving clinical symptoms; scalable; and, thus, may reduce disease burden in those with EDs at low cost.

## Appendix

Multimedia Appendix 1 CONSORT-EHEALTH checklist (V 1.6.1).

## Abbreviations

CBT: cognitive behavioral therapy
ED: eating disorder
EDE-Q: Eating Disorder Examination Questionnaire
ITT: intention-to-treat
MCAR: missing completely at random
mHealth: mobile health
OR: odds ratio
RR: Recovery Record
RR-S: standard RR app
RR-T: tailored version of RR app
